# Density of Green Spaces and Cardiovascular Risk Factors in the City of Madrid: The Heart Healthy Hoods Study

**DOI:** 10.3390/ijerph16244918

**Published:** 2019-12-05

**Authors:** Elena Plans-Beriso, Pedro Gullón, Alba Cebrecos, Mario Fontán, Julia Díez, Mark Nieuwenhuijsen, Manuel Franco

**Affiliations:** 1 Public Health and Epidemiology Research Group, School of Medicine, Universidad de Alcala, 28871 Madrid, Spain; elena.plans@gmail.com (E.P.-B.); alba.cebrecos@uah.es (A.C.); fontan.vela@gmail.com (M.F.); Julia.diez@uah.es (J.D.); mfranco@uah.es (M.F.); 2 Servicio de Medicina Preventiva y Gestión de Calidad, Hospital General Universitario Gregorio Marañón, 28007 Madrid, Spain; 3 Urban Health Collaborative, Drexel Dornsife School of Public Health, Philadelphia, PA 19104, USA; 4 Servicio de Medicina Preventiva, Hospital Universitario Infanta Leonor, 28031 Madrid, Spain; 5 ISGlobal, Center for Research in Environmental Epidemiology (CREAL), 08036 Barcelona, Spain; mark.nieuwenhuijsen@isglobal.org; 6 Department of Biomedicine, Universitat Pompeu Fabra (UPF), 08002 Barcelona, Spain; 7 Centro de Investigación Biomédica en Red de Epidemiología y Salud Pública (CIBERESP), 28029 Madrid, Spain; 8 Department of Epidemiology, Johns Hopkins Bloomberg School of Public Health, Baltimore, MD 21205, USA

**Keywords:** green spaces, cardiovascular risk factors, gender, obesity, hypercholesterolemia, hypertension, diabetes

## Abstract

The aim of this study is to evaluate the relationship between the density of green spaces at different buffer sizes (300, 500, 1000 and 1500 m) and cardiovascular risk factors (obesity, hypertension, high cholesterol, and diabetes) as well as to study if the relationship is different for males and females. We conducted cross-sectional analyses using the baseline measures of the Heart Healthy Hoods study (*N* = 1625). We obtained data on the outcomes from clinical diagnoses, as well as anthropometric and blood sample measures. Exposure data on green spaces density at different buffer sizes were derived from the land cover distribution map of Madrid. Results showed an association between the density of green spaces within 300 and 500 m buffers with high cholesterol and diabetes, and an association between the density of green spaces within 1500 m buffer with hypertension. However, all of these associations were significant only in women. Study results, along with other evidence, may help policy-makers creating healthier environments that could reduce cardiovascular disease burden and reduce gender health inequities. Further research should investigate the specific mechanisms behind the differences by gender and buffer size of the relationship between green spaces and cardiovascular risk factors.

## 1. Introduction

Cardiovascular diseases (CVD) are the leading cause of death worldwide [1,2]. The global number of deaths from CVD has increased globally during the last decade. In fact, in 2016, CVD were responsible for 17.9 million deaths [2], representing one of the major challenges for public health [3]. One of the reasons for this is the increasing prevalence of individual modifiable cardiovascular risk factors, such as obesity, high blood pressure, high cholesterol levels, or diabetes, as well as environmental factors (air pollution, noise, etc.) [4]. In fact, it is estimated that, in Spain, 21.6% of adults are obese [5], 42.6% have hypertension [6], 44.9% high cholesterol [7], and 13,8% diabetes mellitus [8]. Moreover, there is a gender gap in CVD and CVD risk factors; females tend to have a worse risk factor profile compared to males and are more susceptible to risk-factor comorbidity [9].

In the last years, there has been an increasing interest in potential population prevention approaches that could reduce cardiovascular risk factors and, in turn, prevent CVD [10]. Cities present unique opportunities to apply these population prevention approaches, as by definition they are dense, and characterized by substantial man-made components and frequent social interactions [11]. For instance, there is mounting evidence that the availability of parks and other green spaces has benefits for the health and health-related behaviors of urban residents [12,13]. There are different theories that suggest that green spaces affect health through different pathways, such as reducing harm (mitigating exposures to heat, noise, and air pollution), relieving mental and physiologic stress, and promoting healthy activities such as physical activity [14,15].

Previous evidence linked residential green spaces with cardiovascular health and cardiovascular risk factors. There is evidence that a high amount of green spaces is associated with a decrease in cardiovascular mortality [16], a lower hazard of CVD [17], a lower cardiovascular risk [18], and some cardiovascular risk factors, such as obesity [19] and high blood pressure [20]. However, there is no consistent pattern of associations with cardiovascular risk factors, especially in adults, as some studies found green spaces to be associated with a decrease in CVD risk factors while others found no associations [12,13]. Moreover, some studies have suggested that there is a potential effect modification by gender that could change the relationship between green spaces and cardiovascular risk [13,19]. Thus, this study aims to study the relationship between urban green space density and cardiovascular risk factors (obesity, hypertension, high cholesterol, and diabetes) and to study if the relationship is different for males and females.

## 2. Materials and Methods

### 2.1. Study Design and Setting

This study is an observational cross-sectional study aiming to study the relationship between the density of green spaces around the residential location and cardiovascular risk factors in the city of Madrid, Spain. In 2016, Madrid had a population of 3.2 M residents and was divided into 21 districts that housed 128 neighborhoods. Within each neighborhood, there are small geographical administrative units of ~1500 people each, called census sections (*N* = 2415) [21].

### 2.2. Study Population

This study uses the baseline measures of the Heart Healthy Hoods (HHH) cohort. The HHH cohort includes 1720 residents of Madrid that (1) were 40–75 years old in 2017, (2) lived in Madrid (and in the same home address for, at least, one year), (3) were born in Spain or the Andean countries (Ecuador, Peru, Colombia or Bolivia, as that they represent 72.6% of all the South-Americans living in Madrid, and South-Americans are the largest group of migrant residents in Madrid). We excluded potential participants that (a) had previous cardiovascular disease, (b) were institutionalized population, (c) expected to travel outside Madrid more than 3 months per year, (d) were immobilized at home or with terminal or serious conditions that could alter their blood sample values or regular activities, (e) could not answer the telephone questionnaires, (f) planned to move outside Madrid in the following three years.

The sample selection process was carried out in a two-stage process. We first selected 30 primary health care (PHC) centers capturing spatial and sociodemographic variability in the city of Madrid, and then randomized potential participants that met the selection criteria from the PHC physicians’ patient list. Spain’s National Health System is publicly funded, providing universal health care coverage free of charge at the point of use. Every resident has a primary health care physician and a primary health care center assigned to public insurance. The data used for this study was collected through a clinical visit of the participants with their primary health care physician. From the original sample of 2265 potential participants, 1720 attended the clinical visit. In this clinical visit, participants answered a questionnaire with sociodemographic variables, family history of cardiovascular disease and cardiovascular risk factors, quality of life measures, as well as took anthropometric (blood pressure, body mass index) and blood sample tests (fasting blood glucose and low-density-lipoproteins—LDL). The previous diagnosis of cardiovascular risk factors was obtained through their electronic health records. For this study, we excluded participants that did not have complete information on cardiovascular risk factors, anthropometric measures, or blood tests (*N* = 95, 5.5%), leaving a final sample of 1625 participants for the statistical analyses. The 1720 participants that attended the clinical visit had a similar distribution of age, sex, and migration status compared to the original sample of 2265. The HHH study was conducted according to the guidelines laid down in the Declaration of Helsinki and received IRB approval from the Ethics Research Committee of the Madrid Health Care System on 12 May 2015.

### 2.3. Exposure: Density of Green Spaces

We obtained all green spaces land cover categories from the General Urban Plan of the City of Madrid for the year 2016, and we extracted those with a size greater than 0.5 hectares, as a minimum size for doing physical activity [15]. The General Urban Plan of the City of Madrid contains information on official land use categories of all blocks, plots, and spaces in the city of Madrid. We took all green spaces categories available as a land use category, including urban parks as well as other neighborhood green spaces. A detailed classification of all categories used for our definition of green spaces can be found in Supplementary File S1. The land cover distributions and Madrid’s Urban Plan are publicly available at Madrid’s Open Data (https://datos.madrid.es/).

From the General Urban Plan data, we calculated the percentage of green land cover from the population-weighted centroid of the participants’ residence census section. Madrid’s census sections have an average size of 0.2 km^2^, so they are widely used for neighborhood characterization [21]. We used a population-weighted centroid instead of a geometric centroid to avoid locate residents in a non-residential area (likely to be green space) and reduce the risk of misclassification using census measures for the exposure. Thus, we used ArcGIS 10.1 software (v.10.1, ESRI, Redlands, CA, USA) to calculate the percentage of green land cover using different street network buffers (300 m, 500 m, 1000 m and 1500 m) from the population-weighted centroid of each census-section.

### 2.4. Outcome: Cardiovascular Risk Factors

Four individual modifiable cardiovascular risk factors were our main outcome variables: obesity, hypertension, high cholesterol, and diabetes. We classified as obese those participants that had in their electronic health records a diagnosis of obesity, and those participants that had a BMI greater or equal to 30 kg/m^2^, based on the anthropometric measurements [22]. For the latter, we computed the average BMI from the three different measurements that the doctor took during the same clinical visit. For hypertension, we classified as having hypertension those participants previously diagnosed with hypertension (by the electronic health records), those participants in treatment for hypertension and those participants with a mean systolic blood pressure ≥ 140 mmHg or a mean diastolic blood pressure ≥ 90 mmHg after three blood pressure measures during the same clinical visit, following European Society of Cardiology’s recommendations [23]. Hypercholesterolemia was defined as previously diagnosed hypercholesterolemia, cholesterol treatment or LDL greater than 160 mg/dL [24]. We classified as having diabetes those participants with clinical records of diabetes, diabetes treatment or fasting plasma glucose ≥ 126 mg/dL [24].

### 2.5. Covariates

Age, sex, and migration status were self-reported by participants and registered in the clinical visit. To account for the effects of area characteristics, we also adjusted our analyses for the area-level (census section) socioeconomic status (SES) where participants resided in. We used an SES index that includes seven indicators: (1) low education; (2) high education; (3) part-time employment; (4) temporary employment; (5) manual occupational class; (6) average housing prices (per m^2^); (7) unemployment rate [25]. We included population density (number of residents/km^2^) at the census section using annual population data from Padron (a continuous and universal census collected for administrative purposes) [26].

### 2.6. Data Analysis

We conducted an exploratory and descriptive analysis of the exposure, outcomes, and covariates. For continuous variables, we calculated medians and interquartile range; for categorical variables, frequency tables.

To study the relationship between the availability of green spaces within 300, 500, 1000 and 1500 m buffers with cardiovascular risk factors, we estimated odds ratios (ORs) of each cardiovascular risk factor with a set of logistic mixed-effects (also known as multilevel) regression models. Thus, each model included a cardiovascular risk factor as the dependent variable, and the availability of green spaces (at 300, 500, 1000, or 1500 m) as the main explanatory variable (divided into quartiles, where *Q1* represents the greatest availability of green spaces and it is used as the reference value). Separate models were calculated for each density (300, 500, 1000 and 1500 m). All models were adjusted by sex, age, migration status, census-section SES, and population density, and included a random intercept for the census section. Thus, the general formula for all these models is

Logit (odds for risk factor_ij_) = β_0_ + β_1_*density(Q2)_j_ + β_2_*density(Q3)_j_ + β_3_*density(Q4)_j_ + β_4_*age_ij_ + β_5_*sex_ij_ + β_6_*migration_ij_ + β_7_*SES_j_ + β_8_*pop density_j_ + u_j_ + e_ij_

where *i* indexes every participant and *j* every census section. *β*_1,_ *β*_2_, and *β*_3_ are the coefficients for *Q2*, *Q3*, and *Q4* of green spaces density, respectively. *β*_4_*–β*_8_ are the coefficients for the covariates. *u_j_* and *e_ij_* represent the census-section and the individual residual, respectively, both following a normal distribution (0, σ^2^).

We then ran the same models stratified by females and males to obtain specific estimates by gender. In addition to this, to formally obtain an overall statistical *p*-value for effect modification, we ran new models introducing an interaction term between sex and density (in this case, introduced as linear). This way we were able to get a *p*-value for the interaction between sex and density (as linear) instead of an interaction term between each quartile and sex. These models were as follows:

Logit (odds for risk factor_ij_) = β_0_ + β_1_*density(as linear)_j_ + β_2_*age_ij_ +β_3_*sex_ij_ + β_4_*migration_ij_ + β_5_*SES_j_ + β_7_*pop density_j_ + β_8_*density(as linear)_j_*sex_ij_ + u_j_ + e_ij_

Statistical significance was set at *p* = 0.05; thus, all 95% CI that did not include 1 were considered statistically significant. All analyses and plots were conducted with R V3.5.1. Multilevel models were calculated using the *glmer* function in the *lme4* package.

## 3. Results

Table 1 shows the characteristics of the study sample, stratified by quartiles of the percentage of green spaces within a 500 m buffer of the census section. The median age was 56 (IQR = 15), 56.06% of the sample were female, and 19.38% of the participants were born outside Spain. The prevalence of obesity, hypertension, high cholesterol and diabetes was 28.43%, 25.35%, 30.69%, and 8.43%, respectively. Population density and socioeconomic status were higher where participants had a lower density of green spaces.

In the regression models adjusted by all co-variates, we did not find any association between the density of green spaces and obesity and hypertension (Table 2). For high cholesterol, we found an increased odds of having high cholesterol with decreases in the density of green spaces, especially within 300, 500 and 1000 m buffers (only in *Q4*); for instance, living in the quartile with the lowest density of green spaces at 300, 500, and 1000 m (*Q4*) was associated with an increased odds of high cholesterol of 46% (95% CI: 5% to 103%), 47% (95% CI: 5% to 106%), and 55% (95% CI: 10% to 118%), respectively. There was a relationship between the density of green spaces within a 500 m buffer and diabetes (only in *Q2*); the odds of having diabetes increased by 67%, in *Q2* (95% CI: 1% to 176%); however, it was not significant for *Q3* and *Q4*.

**Figure 1:**
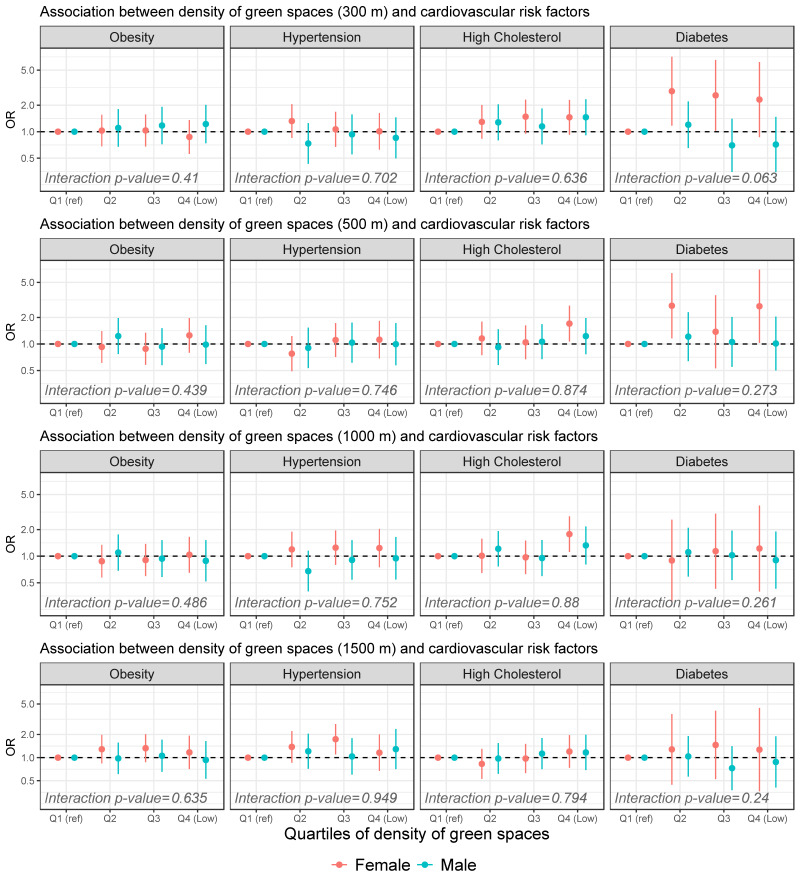
Association between the density of green spaces around participants’ residences (300, 500, 1000 and 1500 m buffers) and cardiovascular risk factors in Madrid (*N* = 1625). Mixed-effects logistic regression models were stratified by females (red) and males (blue) and adjusted by age, migration status, population density, and area-level socioeconomic status. Rows represent the different density of green spaces around the home. From top to bottom: 300 m, 500 m, 1000 m and 1500 m. Columns represent each of the cardiovascular risk factors, from left to right: obesity, hypertension, high cholesterol, and diabetes. The interaction *p*-values represent the interaction between sex and the density of green spaces (as linear instead of categorical).

In the gender-stratified models, we found that female participants showed higher OR than males in the relationship between green spaces and cardiovascular risk factors (Figure 1 and Supplementary Table S2). For obesity, there were no significant associations neither in males or females and the interaction was non-significant; however, within the 1500 m buffer, there was a non-significant increase in odds of having obesity with decreasing density, but only in female participants. In the case of hypertension, we found a relationship between the density of green spaces within a 1500 m buffer and an increased odds of hypertension, but only in females. For instance, females living in *Q3* of green space density within a 1500 m buffer had an increased odds of 73% (95% CI: 10% to 173%) for hypertension. Despite this, the interaction term between density and sex was non-significant for all densities in hypertension. For high cholesterol, the associations observed in the non-stratified models were only still present in the models with females. Thus, there were increased odds for high cholesterol for females living in the areas with a low density of green spaces within 500 and 1000 m buffers. For diabetes, we found an association between 300 m and 500 m buffers of green space density and diabetes only in females (interaction term between sex and density for 300 m *p* = 0.06; for 500 m *p* = 0.27); for instance, females living in *Q2*, *Q3*, and *Q4* of lower green space density (300 m) had an OR of 2.88 (95% CI: 1.17 to 7.10), 2.59 (95% CI: 1.02 to 6.52), and 2.32 (95% CI: 0.86; to 6.18), respectively.

## 4. Discussion

### 4.1. Key Findings

In this study, we found a moderate association between density of green spaces around participant’s location (within 300, 500, 1000 and 1500 m buffers) and hypertension, high cholesterol, and diabetes, but not for obesity; particularly, females living in areas of lower green space density had greater odds for specific cardiovascular risk factors (hypertension, high cholesterol, and diabetes) compared to those that live in the highest density areas (*Q1*). These results are relevant because they deepen the knowledge on the relationship between green spaces and cardiovascular health in the specific case of the city of Madrid, and they open new questions regarding the gender dimension in the studies of green spaces and cardiovascular health.

### 4.2. Comparison with Previous Studies and Mechanisms

Despite the lack of evidence of our results for obesity, other studies found an inverse relationship between the increased availability of green spaces and obesity [27]. We hypothesize two main reasons that could explain why we did not find an association between the density of green spaces and obesity. First, we did not take into account any measure of the quality of green spaces. If we hypothesize that green spaces might prevent obesity through physical activity, these spaces should be designed for that. In fact, there are studies that suggest that certain characteristics of green spaces such as size, sports facilities, quality of paths, and a safe environment might be relevant for physical activity within green spaces [28,29]; for instance, Kaczynski et al. [28] observed that the quality of park trails and the number of facilities and amenities in the park were associated with park-based physical activity. Second, green spaces are usually located in areas with less population and retail density due to lack of space, and these areas might discourage walking and physical activity as they are less walkable [30]. Despite adjusting by population density as a proxy of walkability, we did not test effect modification by population density/walkability; meaning that we did not assess if the relationship between green spaces and obesity is stronger or weaker in areas with high walkability.

Females living in areas with a lower density of green spaces (at 1500 m) had higher odds for hypertension after adjusting by individual and neighborhood characteristics. Previous studies support the idea that greater availability of green spaces around home reduces blood pressure in adults [31,32], especially by harm reduction (e.g., exposure to noise or air pollution) [33], psychological and physiological stress alleviation, increased social cohesion, or physical activity [15]. Similarly, we found a relationship between green spaces and hypercholesterolemia, consistent with other previous studies [27,31,34], that suggested that green spaces could reduce cholesterol levels via physical activity, harm reduction or psychosocial pathways. However, we did not test the mediating pathways through which green spaces might reduce blood pressure or cholesterol levels. Lastly, we also found an association of green spaces density (300 m and 500 m) and diabetes in females, consistent with other studies [35,36]. However, mechanisms behind the association between green spaces and diabetes are not so clear; for instance, Dalton et al. [35] found that physical activity did not mediate the relationship between green spaces and diabetes, and Bodicoat et al. [36] did not find that physical activity and other risk factors explained this association.

### 4.3. Effect of Gender and Buffer Size

Overall, we only found a relationship between green spaces and cardiovascular risk factors (hypertension, high cholesterol, and diabetes) in female participants. A previous systematic review suggested that the association of green spaces and most mortality outcomes was greater in women and that more research was needed on the different effects of green spaces in males and females [12]. Gender differences in the relationship between green spaces and health have been found in other studies. In fact, previous studies have found stronger effects of green spaces on health for women [19,37], while others not [38]. Indeed, one study found an interaction between age, gender, and the association between green spaces with health; whilst the benefit of greater local green space for men was apparent primarily in early to mid-adulthood, the benefit for women occurred later in life, in their mid−40 s and older [39] (similar to our sample). One possible explanation to the gender differences is that males and females have different social use and perception of green spaces; for instance, males are more likely than females to use green spaces for physical activity [40] and females may not use green spaces if they perceive them as unsafe [41]; in fact, a systematic review with systematic social observation found that, when using green spaces, females seem to be more sedentary than male [42]. However, this would explain only the physical activity pathway, not alternative pathways such as reduced exposure to air pollution and noise. Moreover, our results go against this hypothesis as they go in the opposite direction (green spaces might have more benefit for females). An alternative hypothesis might be that women have stronger benefits of green spaces as they spent more time in the surrounding neighborhood as they may feel socially responsible for housekeeper activities; in fact, a previous study found that the relationship between greenspace and health was stronger for housewives [43]. Future studies should clarify the pathways through which gender interacts in the association between green spaces and health. Unexpected, most of the interaction terms were non-significant. However, this could be because the interaction term between density (as linear) and sex assumed a linear relationship between exposure and outcome (which is not always the case in this study). Despite this fact, we observed that the sub-group analysis that most OR (especially in diabetes) were higher for women. We should also consider that the use of stratified models might have caused a loss in statistical power to detect more statistically significant associations by gender.

Another important key result of our study is the different buffer effects depending on the risk factor. We found that the relationship of green spaces density and hypertension was only present in larger buffers (1000 and 1500 m); while the relationship of green spaces and high cholesterol or diabetes was stronger for smaller buffers (300 and 500 m). Some studies suggested that larger buffers have stronger associations with health outcomes [37] as well as they might better represent distances that people are willing to do for visiting parks and green spaces [43]; however, most of the studies did not find evidence of different effects by buffer size, and suggests that buffer size selection might depend on the context of cities (density, spatial configuration, etc.) [12]. It should be taken into account that we used the population-weighted centroid as the start of our buffers instead of the participants’ exact residence, and smaller buffers (300 m) might be subjected to misclassification.

### 4.4. Strengths and Limitations

This study presents several strengths. Firstly, we were able to test different buffer sizes, providing more insights into how different sizes might be more important for different pathways between green spaces and health. Secondly, we used the street network instead of Euclidean buffers to calculate distances and more accurately adjust the true density of green spaces. Thirdly, despite the common use of satellite-derived land cover distributions and surrounding greenness, such as the Normalized Difference Vegetation Index (NDVI) [14], land use data from the Urban Plan of the City of Madrid is an official tool used by urban planners and policymakers in Madrid, so it might be relevant for policy change. Lastly, we were able to combine two different measures for defining participants with cardiovascular risk factors: practitioners’ previous diagnoses (by electronic health records) and anthropometric measures (blood pressure and BMI) obtained in a clinical visit, which could reduce information bias compared to self-reported measures or the use of electronic health records alone.

We are aware that this study has several limitations. This is a cross-sectional study, which does not allow us to claim causality of our results. Besides, we have not taken into account the amount of time that each person has been visiting green spaces, so in future studies, just like Paquet et al. [44], we suggest comparing the association between the group of residents with different time exposure to green spaces, even though visiting green spaces is not the only pathway that could explain this association (as said above, other hypothetical pathways might be, for instance, the reduction of air pollution, heat, and noise). We did not include measures of quality of green spaces (as the quality of trails, safety, etc.), which might influence the use of these spaces. Our exposure measures (density of green spaces) were calculated from the census-section population-weighted centroid instead of the exact residence location, which could lead to a misclassification in the exposure; however, census sections are small spatial units (average size: 0.2 km^2^), thus we don’t expect a significant misclassification of residence location. Moreover, the use of a population-weighted centroid prevents us to locate the centroid in a non-residential area (which might be a green area). Our stratified models by male and female might have caused a loss in statistical power to detect more statistically significant associations by gender. Lastly, we were not able to test for the specific pathways through which green spaces are connected with cardiovascular risk factors; future studies should study the pathways between green spaces and cardiovascular risk factors with a gender perspective.

## 5. Conclusions

We found a moderate protective relationship between green space density and several cardiovascular risk factors in female participants; hypercholesterolemia, hypertension, and diabetes, but not for obesity. We found different effects for females and males and for different buffer distances: there was a stronger association with hypertension when using larger buffers, while small buffers showed stronger associations with hypercholesterolemia and diabetes. Future studies should further study the mechanisms of this association, as well as the different effects by buffer size and gender so policy-makers can design urban policies to prevent cardiovascular risk taking into account the differences in the effects by gender.

## Figures and Tables

**Table 1:** Characteristics of the Heart Healthy Hoods (HHH) cohort study sample, stratified by quartiles of % of green space density within 500 m buffer of participants’ census section centroid (*N* = 1625).

				Quartiles of Green Space Density (within 500 m)						
Individual Characteristics	Total		Q1 (High)		Q2		Q3		Q4 (Low)	
Age ^1^ (years)	56	15	56	16	56	14.75	56	14.75	56	15
Sex 2 (female)	911	56.06%	234	57.64%	230	56.65%	225	55.42%	222	54.55%
Migration status ^2^	315	19.38%	80	19.70%	84	20.69%	81	19.95%	70	17.20%
Obesity ^2^	462	28.43%	120	29.56%	123	30.30%	110	27.09%	109	26.78%
Hypertension ^2^	412	25.35%	105	25.86%	93	22.91%	110	27.09%	104	25.55%
High cholesterol ^2^	502	30.89%	116	28.57%	119	29.31%	121	29.80%	146	35.87%
Diabetes ^2^	137	8.43%	28	6.90%	44	10.84%	32	7.88%	33	8.11%
Population density ^1^ (pop/km^2^)	30,784	23,067	27,333	28,610	28,391	20,360	29,604	21,766	36,840	20,643
Socioeconomic status index ^1^	−0.37	1.24	−0.40	0.81	−0.44	0.90	−0.42	0.94	0.17	1.93

^1^ Median and IQR. ^2^ N and %.

**Table 2:** Association between the density of green space around the participants’ residence (300, 500, 1000 and 1500 m buffers) and cardiovascular risk factors in Madrid (*N* = 1625). Mixed-effects logistic regression models adjusted by age, sex, migration status, population density, and area-level socioeconomic status.

	300 m		500 m		1000 m		1500 m	
Green Spaces	OR ^1^	CI 95% ^2^	OR	CI 95%	OR	CI 95%	OR	CI 95%
	Obesity							
Q1 (ref)	1 (ref)		1 (ref)		1 (ref)		1 (ref)	
Q2	1.05	(0.76–1.43)	1.05	(0.77–1.44)	0.95	(0.69–1.29)	1.14	(0.84–1.56)
Q3	1.08	(0.79–1.48)	0.89	(0.65–1.22)	0.91	(0.67–1.25)	1.20	(0.88–1.65)
Q4 (Low)	1.00	(0.72–1.38)	1.09	(0.78–1.52)	0.95	(0.67–1.34)	1.05	(0.73–1.52)
	Hypertension							
Q1 (ref)	1 (ref)		1 (ref)		1 (ref)		1 (ref)	
Q2	0.98	(0.71–1.37)	0.84	(0.60–1.18)	0.89	(0.64–1.25)	1.32	(0.93–1.85)
Q3	1.01	(0.72–1.40)	1.06	(0.76–1.47)	1.05	(0.76–1.46)	1.38	(0.98–1.95)
Q4 (Low)	0.92	(0.65–1.30)	1.03	(0.72–1.46)	1.06	(0.74–1.53)	1.20	(0.81–1.79)
	High Cholesterol							
Q1 (ref)	1 (ref)		1 (ref)		1 (ref)		1 (ref)	
Q2	1.28	(0.93–1.77)	1.05	(0.76–1.44)	1.11	(0.80–1.53)	0.90	(0.65–1.24)
Q3	1.32	(0.95–1.83)	1.06	(0.77–1.46)	0.97	(0.70–1.33)	1.04	(0.75–1.43)
Q4 (Low)	1.46	(1.05–2.03)	1.47	(1.05–2.06)	1.55	(1.10–2.18)	1.20	(0.83–1.71)
	Diabetes							
Q1 (ref)	1 (ref)		1 (ref)		1 (ref)		1 (ref)	
Q2	1.61	(0.98–2.64)	1.67	(1.01–2.76)	1.01	(0.62–1.67)	1.15	(0.70–1.87)
Q3	1.15	(0.68–1.95)	1.15	(0.68–1.96)	1.05	(0.64–1.72)	0.97	(0.58–1.61)
Q4 (Low)	1.09	(0.63–1.90)	1.44	(0.82–2.52)	0.99	(0.56–1.75)	1.00	(0.55–1.83)

^1^ OR, Odds ratio. ^2^ CI, Confidence interval.

## Supplementary Materials

The following are available online at https://www.mdpi.com/1660-4601/16/24/4918/s1, File S1: Types of green spaces according to General Urban Plan of the city of Madrid, Table S2: Association between density of green space around the participants’ residence (300, 500, 1000 and 1500 m buffers) and cardiovascular risk factors in Madrid.
